# CREB regulates spine density of lateral amygdala neurons: implications for memory allocation

**DOI:** 10.3389/fnbeh.2013.00209

**Published:** 2013-12-20

**Authors:** Derya Sargin, Valentina Mercaldo, Adelaide P. Yiu, Gemma Higgs, Jin-Hee Han, Paul W. Frankland, Sheena A. Josselyn

**Affiliations:** ^1^Program in Neurosciences and Mental Health, Hospital for Sick ChildrenToronto, ON, Canada; ^2^Department of Psychology, University of TorontoToronto, ON, Canada; ^3^Department of Physiology, University of TorontoToronto, ON, Canada; ^4^Institute of Medical Science, University of TorontoToronto, ON, Canada; ^5^Laboratory of Neural Circuit and Behavior, Department of Biological Sciences, KAIST Institute for the BioCentury, Korea Advanced Institute of Science and TechnologyDaejeon, Korea

**Keywords:** CREB, amygdala, fear memory, dendritic spines, viral vector

## Abstract

Neurons may compete against one another for integration into a memory trace. Specifically, neurons in the lateral nucleus of the amygdala with relatively higher levels of cAMP Responsive Element Binding Protein (CREB) seem to be preferentially allocated to a fear memory trace, while neurons with relatively decreased CREB function seem to be excluded from a fear memory trace. CREB is a ubiquitous transcription factor that modulates many diverse cellular processes, raising the question as to which of these CREB-mediated processes underlie memory allocation. CREB is implicated in modulating dendritic spine number and morphology. As dendritic spines are intimately involved in memory formation, we investigated whether manipulations of CREB function alter spine number or morphology of neurons at the time of fear conditioning. We used viral vectors to manipulate CREB function in the lateral amygdala (LA) principal neurons in mice maintained in their homecages. At the time that fear conditioning normally occurs, we observed that neurons with high levels of CREB had more dendritic spines, while neurons with low CREB function had relatively fewer spines compared to control neurons. These results suggest that the modulation of spine density provides a potential mechanism for preferential allocation of a subset of neurons to the memory trace.

## Introduction

The cAMP Responsive Element Binding Protein (CREB) is an activity regulated transcription factor that modulates the transcription of genes with cAMP responsive elements (CRE) located in their promoter regions. Early research in *Aplysia* (Dash et al., 1990; Kaang et al., 1993; Bartsch et al., 1995) and *D. melanogaster* (Yin et al., 1994, 1995; Perazzona et al., 2004) first implicated CREB in memory formation. Since that time, the important role of CREB in memory has been shown across a variety of species from *C. elegans* (Kauffman et al., 2010; Lau et al., 2013) to rats (Guzowski and McGaugh, 1997; Josselyn et al., 2001), mice (Bourtchuladze et al., 1994; Kida et al., 2002; Pittenger et al., 2002; Gruart et al., 2012) and humans (Harum et al., 2001) (for review, see Josselyn and Nguyen, 2005) but see Balschun et al. (2003). For instance, we (Han et al., 2007), and others (Zhou et al., 2009; Rexach et al., 2012) previously showed that increasing CREB function in a small portion of lateral amygdala (LA) neurons (roughly 8–10% of LA principal neurons) was sufficient to enhance auditory fear memory. Moreover, we observed that LA neurons with relatively higher CREB function at the time of training were preferentially included, whereas neurons with lower CREB function were excluded, from the subsequent LA fear memory trace (Han et al., 2007, 2009). Conversely, disrupting CREB function by expressing a dominant negative version of CREB (CREB^S133A^) in a similar small percentage of LA neurons did not affect auditory fear memory, likely because the neurons expressing CREB^S133A^ were largely excluded from the memory trace. Furthermore, post-training ablation (Han et al., 2009) or silencing (Zhou et al., 2009) of neurons overexpressing CREB disrupted subsequent expression of the fear memory, confirming the importance of these neurons. Together, these data suggest that neurons with high levels of CREB at the time of training are preferentially allocated to the memory trace because they somehow outcompete their neighbors (Won and Silva, 2008).

CREB is a ubiquitous transcription factor implicated in many diverse cellular processes in addition to memory formation, including regulation of proliferation, survival, apoptosis, differentiation, metabolism, glucose homeostasis, spine density, and morphology (Bourtchuladze et al., 1994; Murphy and Segal, 1997; Silva et al., 1998; Mayr and Montminy, 2001; Lonze et al., 2002; Wayman et al., 2006; Aguado et al., 2009; Altarejos and Montminy, 2011). Which of these CREB-mediated processes is/are important for memory allocation? Here we investigated one CREB-mediated process, the regulation of spine density and morphology.

Dendritic spines are small, highly motile structures on dendritic shafts which provide flexibility to neuronal networks. As an increase in the synaptic strength between neurons is thought to underlie memory formation (Bailey and Kandel, 1993; Bailey et al., 1996) and the majority of excitatory synapses occur on dendritic spines (Harris and Stevens, 1988, 1989; Farb et al., 1992), it has long been thought that dendritic spines serve as storage sites for synaptic strength, an idea first proposed by Santiago Ramón y Cajal over 100 years ago (Cajal, 1995). In this way, the growth and re-structuring of dendritic spines is thought to be crucial for memory formation.

A role for CREB in spine formation was first reported by Murphy and Segal (1997) who showed that estradiol treatment increased both levels of activated (phosphorylated) CREB and spine density in cultured hippocampal neurons. CREB was subsequently shown to regulate spine morphology in hippocampal neurons both in organotypic culture (Impey et al., 2010) and *in vivo* (Marie et al., 2005), as well as in visual cortex principal neurons (Suzuki et al., 2007). The new spines formed following overexpression of CREB may contain silent synapses (NMDA receptors only), suggesting that they may be “primed” for incorporation into future memory circuits (Marie et al., 2005). Consistent with this, increasing CREB function in hippocampal CA1 principal neurons was sufficient to restore both the decrease in spine density and spatial memory in a mouse model of Alzheimer's disease (Yiu et al., 2011).

We previously reported that neurons with increased CREB at the time of training are selectively allocated to a fear memory trace and a variety of evidence shows that increasing CREB function increases spine density. Therefore, we investigated whether neurons with increased CREB at the time of training also have an increase in dendritic spine density, thereby providing a potential mechanism of the preferential allocation of these neurons to the memory trace.

## Materials and methods

### Mice

Adult male F1 hybrid (C57 BL/6NTac × 129S6/SvEvTac) mice were used for all experiments. This genetic background has been used extensively in behavioral studies and are well characterized (Silva et al., 1997). Mice were group housed (2–5 mice per cage) on a 12 h light/dark cycle and provided with food and water *ad libitum*. All experimental procedures were conducted in accordance with the guidelines of the Canadian Council on Animal Care (CCAC) and the National Institutes of Health (NIH) and approved by the Animal Care Committee at the Hospital for Sick Children.

### HSV vectors

Neurotropic replication-defective herpes simplex viral (HSV) vectors were used to manipulate CREB function in individual LA principal neurons. Wild-type or dominant negative CREB^S133A^ cDNAs were cloned into the HSV amplicon under the control of the constitutive promoter for the HSV immediate early gene IE4/5. These vectors co-expressed GFP which was driven by CMV promoter [HSV-p1005; Russo et al., 2009]. In this vector therefore, the GFP protein is not fused to CREB and may thus fill the infected cell. As a control, we used HSV vector expressing GFP alone. HSV virus was packaged using a replication-defective helper virus as previously described (Josselyn et al., 2001; Barrot et al., 2002; Carlezon and Neve, 2003; Han et al., 2008; Vetere et al., 2011; Cole et al., 2012). Virus was purified on a sucrose gradient, pelleted and resuspended in 10% sucrose. The average titer of the virus stocks was typically 4.0 × 10^7^ infectious units/ml.

### Surgery

Mice were pretreated with atropine sulfate (0.1 mg/kg, i.p.), anesthetized with chloral hydrate (400 mg/kg, i.p.) and placed in a stereotaxic frame. Skin was retracted and holes were drilled in the skull above the LA (anteroposterior = −1.4, mediolateral = ±3.4, ventral = −5.0 mm from bregma) according to (Paxinos and Franklin, 2001). Viral vector was microinjected through glass micropipettes connected via polyethylene tubing to a microsyringe (Hamilton, Reno, NV) at a rate of 0.1 μl/min. Micropipettes were left in place an additional 10 min following microinjection to ensure diffusion of vector. For behavior analysis, a volume of 1.5 μl and for spine analysis, a volume of 1.0 μl was microinjected bilaterally at a rate of 0.1 μl/min. Micropipettes were slowly retracted, the incision site closed and mice were treated with analgesic (ketoprofen, 5 mg/kg, s.c.). Three d following surgery, at a maximal transgene expression for HSV vector system (Josselyn et al., 2001; Barrot et al., 2002; Vetere et al., 2011; Cole et al., 2012), mice were either fear conditioned or perfused for dendritic spine analysis.

### Auditory (tone) fear conditioning

During training, mice were placed in a Med Associates (St. Albans, VT) Plexiglas and metal chamber (24 × 30 × 21 cm, context A; Cxt A) located in a soundproof room. After 2 min, a tone (2800 Hz, 30 s, 85 dB) that co-terminated with a footshock (2 s, 0.4 mA) was presented. Mice remained in the chamber for an additional 30 s and then returned to the homecage. Testing for auditory fear memory occurred 24 h later by placing mice in a novel context (context B; Cxt B) and 2 min later, presenting the tone previously paired with footshock for 3 min. The percentage of time mice spent freezing (the cessation of all movement except respiration) before and during the tone was measured using an automated system (Actimetrics) and was used as our index of memory. Immediately after testing, mice were deeply anesthetized and perfused.

### Immunohistochemistry

To visualize the number and morphology of dendritic spines in the neurons we infected, we took advantage of the GFP expressed by all viral vectors. We amplified the GFP signal using an antibody directed against GFP. 72 h after surgery, mice were deeply anesthetized using chloral hydrate (400 mg/kg, i.p.) and transcardially perfused with 0.1 M PBS followed by 4% paraformaldehyde (PFA). Brains were post-fixed overnight in 4% PFA and transferred to 30% sucrose for cryoprotection. Coronal (50 μm) sections were prepared and immunohistochemistry for GFP was performed. Free-floating sections were incubated in blocking solution (0.1% BSA, 5% NGS, 0.2% Triton-X-100 in 0.1 M PBS) for 1 h and labeled with anti-GFP rabbit polyclonal antibody (1:500, Invitrogen) overnight at 4°C. Following PBS washes, sections were incubated with goat anti-rabbit Alexa 488 (1:500, Invitrogen) for 2 h at room temperature. Sections were washed with PBS, mounted on gelatin-coated slides and coverslipped using Vectashield Hardmount with DAPI (Vector Laboratories).

### Confocal analysis

GFP-positive LA neurons (neurons infected by viral vectors) were first identified using a 10x objective (LSM 710, Zeiss). Infected neurons were included in the subsequent spine analysis if (i) cell body was not damaged; (ii) dendritic projections remained within the LA; (iii) neurons could clearly be identified without interference from neighboring infected cells; (iv) neurons had first, second, and third order branches. Fourth order branches were not included in the analysis as they often appeared truncated in our 50 μm sections. Selected GFP-positive neurons were imaged using a 100x oil-immersion objective. Z series were obtained by imaging serial confocal planes at 0.25 μm intervals. Dendrites and spines were traced manually from the image stacks using Neurolucida software and analyzed with Neurolucida Explorer (MBF version 9).

#### Dendritic morphology

Image analysis was performed by two researchers unaware of the treatment condition of the mouse. Dendrites were traced. The first dendritic process emanating from the cell body was defined as the primary (first order) branch. Subsequent branches that bifurcated from the first branch order were designated as second order branches, and so forth. Truncated branches or those that did not remain within the image window were excluded from subsequent analysis.

#### Spine morphology

Dendritic spines were defined as small protrusions connected to the dendritic shaft (Feldman and Dowd, 1975). Spines show a distinct morphology and vary in length from 0.5–4 μm (Peters and Kaiserman-Abramof, 1970; Horner and Arbuthnott, 1991). Therefore, we analyzed all dendritic protrusions that were less than or equal to 4 μm in length (Horner and Arbuthnott, 1991). Because this method has been shown to produce reliable results (Horner and Arbuthnott, 1991), no attempt was made to introduce a correction factor for hidden spines. Spines were counted and spine density was calculated as the number of spines on a branch divided by the length of the branch. Spine length was defined as the distance between the spine tip and the base of the spine. Spine head diameter was identified as the maximum width of the spine head (see Figure 2B).

### Statistical analysis

Data were analyzed with 1 or 2-Way analyses of variance (ANOVAs) using Statistica (Statsoft) software. For the auditory fear conditioning data, we analyzed the percentage of time spent freezing to before (2 min) and during (3 min) the tone. For dendritic and spine morphological analysis, data were first averaged by branch order per cell, then by animal and finally by vector group (GFP, CREB, or CREB^S133A^). Newman-Keuls *post-hoc* tests were performed where appropriate. To protect against potential type 1 errors resulting from multiple comparisons of 5 different measures of neuronal morphology (i.e., spine density, spine length, spine head diameter, dendrite length, and dendrite volume), we also performed a Bonferroni correction (corrected α = 0.01). All significant main effects remained significant after correction.

## Results

### Increasing CREB in a small portion of LA neurons enhances memory formation while expressing the dominant negative version of CREB has no effect

**Figure 1:**
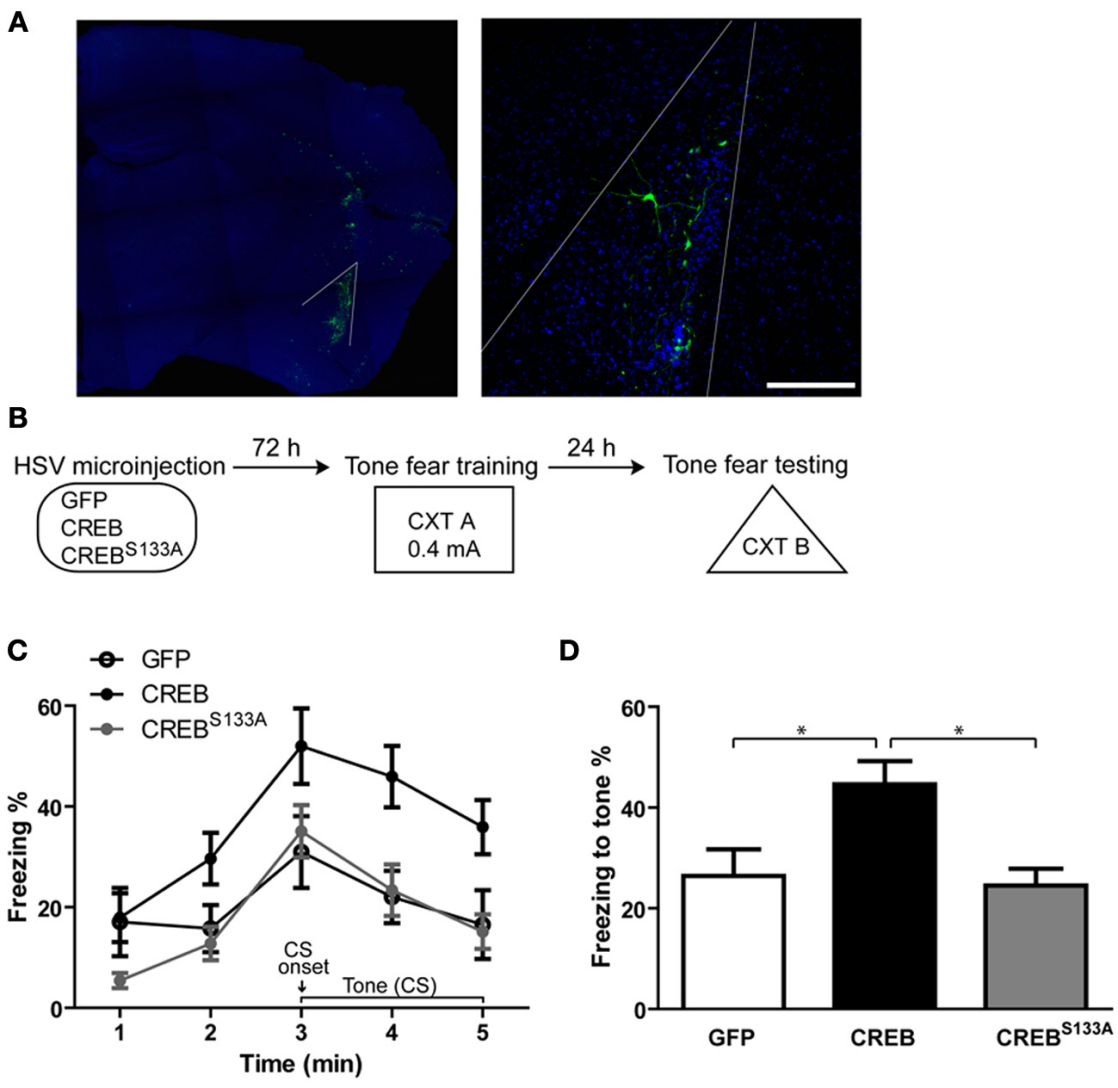
**Overexpressing CREB in LA neurons enhances, while dominant negative CREB^S133A^ does not affect, auditory fear memory. (A)** Visualizing neurons infected with viral vectors. Left: Outline of the LA. Maximum intensity projection is shown. Right: LA principal neurons expressing GFP 72 h following viral vector microinjection (nuclei stained with DAPI, infected neurons visualized with anti-GFP antibody). 0.25 μm optical section is shown. Scale bar, 100 μm. **(B)** Experimental design. Auditory (tone) fear conditioning was conducted 72 h after HSV microinjection. Mice were placed in CXT A and presented with a tone (30 s) that co-terminated with a footshock (0.4 mA). Memory was assessed 24 h later in CXT B. **(C)** Mice microinjected with CREB vector (*n* = 10) showed increased freezing during (but not before) subsequent presentation of the tone compared to mice microinjected with GFP (*n* = 12) or CREB^S133A^ (*n* = 11) vectors. **(D)** Mice overexpressing CREB in LA neurons showed enhanced fear memory for the tone indicated by higher freezing levels during the 3–min tone, compared to mice with GFP or CREB^S133A^ vectors. Data represent mean ± s.e.m. ^*^p < 0.05.

We first confirmed the effects of manipulating CREB function in a small portion (~8–10%) of LA neurons on the formation of tone fear memory by microinjecting HSV vectors encoding GFP, CREB or dominant-negative CREB (CREB^S133A^) into the LA of adult mice 3 d before fear conditioning (see Figure 1A). During training (Cxt A), mice received a single tone (conditioned stimulus, CS) footshock (0.4 mA) (US) pairing that did not induce ceiling levels of freezing. Tone fear memory was assessed 24 h after training. Mice were placed in a novel context (Cxt B) and 2 min later the tone was presented for 3 min (Figure 1B). Consistent with our earlier findings (Han et al., 2007, 2009) and those of other research groups (Zhou et al., 2009; Rexach et al., 2012), increasing CREB levels in a small portion of LA neurons enhanced tone fear memory, while disrupting CREB function by microinjecting CREB^S133A^ vector had no effect on fear memory (Figures 1C,D). These results were supported by a *Vector* (GFP, CREB, CREB^S133A^ vector) × Time (5 min) ANOVA showing significant main effects of *Vector* [*F*_(2, 28)_ = 6.8, *p* = 0.004] and *Time* [*F*_(4, 112)_ = 16.5, *p* ≤ 0.0001] but no *Vector* × *Time* interaction [*F*_(8, 112)_ = 6.8, *p* = 0.32]. A subsequent One-Way ANOVA performed on freezing during the entire CS presentation showed a significant effect of Vector [*F*_(2, 28)_ = 7.1, *p* = 0.003], as mice microinjected with CREB vector froze significantly more than mice with GFP (*p* = 0.006) or CREB^S133A^ vector (*p* = 0.004), which did not differ from each other (*p* = 0.83) (Newman-Keuls *post-hoc*) (Figure 1D). Importantly, when first placed in Cxt B, mice generally showed little freezing before the tone was presented and baseline levels of freezing in CREB or CREB^S133A^ groups did not differ from the GFP group (*p* = 0.18, *p* = 0.19 respectively). We next examined a possible mechanism underlying this preferential recruitment to the memory trace.

### CREB modulates dendritic spine density of LA neurons

**Figure 2:**
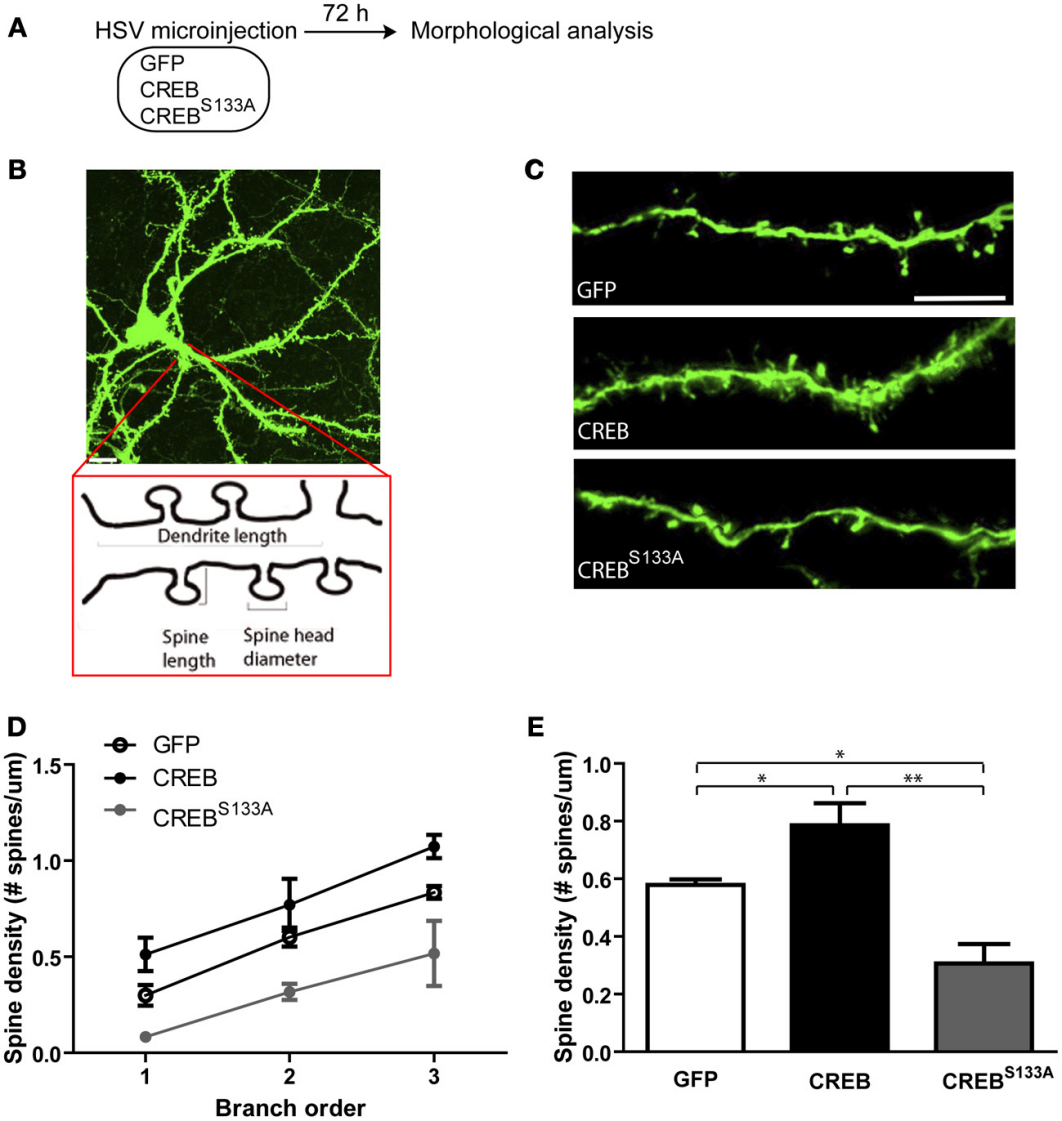
**CREB modulates dendritic spine density in LA neurons. (A)** Experimental design. The morphology of infected LA neurons was analyzed 72 h after mice were microinjected with GFP, CREB or, CREB^S133A^ vectors (at the same time-point as training occurred in Figure 1). Mice remained in the homecage after microinjection and were not fear conditioned. **(B)** Schematic representation of a dendritic segment showing the parameters analyzed (dendrite length, spine length, spine head diameter and spine density). Scale bar, 10 μm. **(C)** Representative dendritic segments of LA neurons from mice microinjected with GFP, CREB, or CREB^S133A^ vector. Scale bar, 5 μm. **(D)** Dendritic spine density shown at each branch order and **(E)** across all branches is increased in neurons with CREB vector (*n* = 6) and decreased in neurons with CREB^S133A^ vector (*n* = 5) when compared to neurons with GFP vector (*n* = 7). Data represent mean ± s.e.m. ^*^p < 0.05. ^**^p < 0.001.

CREB is a ubiquitous transcription factor that has been implicated in many cellular processes, including regulating dendritic spine density. We hypothesized that neurons may be recruited to the memory trace based on their relative spine density, and examined whether neurons infected with CREB vector show greater dendritic spine density *at the time of training* than neurons infected with CREB^S133A^ or Control GFP vector. We microinjected a separate co-hort of mice with GFP, CREB, or CREB^S133A^ vector as above but did not train these mice. Instead, 72 h following surgery (at a time when they would have received auditory fear conditioning) we removed their brains and examined spine density (Figure 2A).

There are two major neuronal cell populations in LA: pyramidal glutamatergic projection neurons and local circuit γ-aminobutyric acid (GABA)-ergic interneurons (McDonald, 1984). Glutamatergic pyramidal-like principal neurons comprise the majority (85–90%) (McDonald, 1992; Sah et al., 2003) and can be visually identified according to the shape of their somata. Thus, we identified infected neurons as LA principal neurons based on their pyramidal shaped somata. In mice microinjected with CREB vector, infected neurons showed higher spine density compared to infected neurons in mice microinjected with Control (GFP-only) vector. In contrast, CREB^S133A^-infected neurons showed lower spine density than control neurons. This pattern of results was observed across branch order (Figures 2C,D). A *Vector* (GFP, CREB, CREB^S133A^) by *Branch order* (3) repeated-measures ANOVA showed significant main effects of *Vector* [*F*_(2, 15)_ = 16.8, *p* ≤ 0.0001] and *Branch order* [*F*_(2, 30)_ = 37.2, *p* ≤0.0001] but no significant interaction between *Vector* × *Branch order* [*F*_(4, 30)_ = 0.2, *p* = 0.90]. *Post-hoc* Newman-Keuls analysis on the significant main effects revealed that neurons with CREB vector had significantly greater spine density across branch orders compared to neurons infected with GFP (*p* = 0.02) or CREB^S133A^ (*p* = 0.0002) vectors (Figures 2C,E), while neurons with CREB^S133A^ vector had lower spine density across branches relative to those expressing GFP only (*p* = 0.004) (Figures 2C,E). It is important to note that these changes in spine density occurred even though all mice were maintained in the homecage (and therefore, these changes in spine density cannot be attributed to fear conditioning).

**Figure 3:**
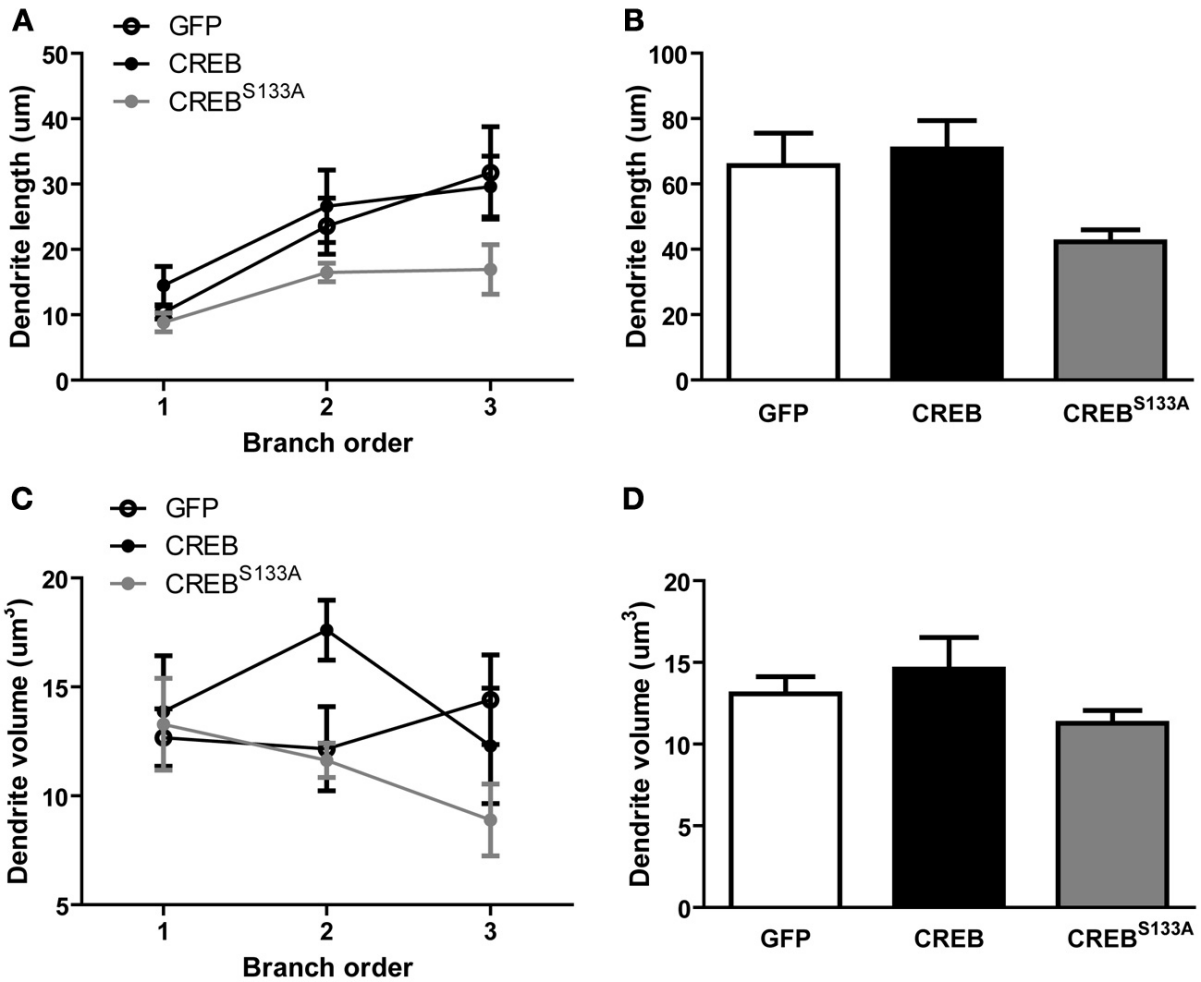
**CREB does not affect dendritic length and volume. (A)** Dendritic length per branch order and **(B)** across all branches was comparable between LA neurons overexpressing GFP (*n* = 7), CREB (*n* = 6), or CREB^S133A^ (*n* = 5) vectors. **(C)** Dendrite volume per branch order and **(D)** across all branches did not differ between LA neurons overexpressing GFP (*n* = 7), CREB (*n* = 6), or CREB^S133A^ (*n* = 5) vectors.

Importantly, dendritic length per branch (Figure 3A) or total dendritic length did not appear to differ between vectors (Figure 3B). This observation was supported by repeated measures ANOVA showing no significant effect of *Vector* [*F*_(2, 15)_ = 2.8, *p* = 0.09] or interaction of *Vector* × *Branch order* [*F*_(4, 30)_ = 0.7, *p* = 0.60], but a significant main effect of *Branch order* [*F*_(2, 30)_ = 10.9, *p* = 0.0003]. Therefore, dendritic length increased with increasing branch order, but this was not changed by CREB manipulation (Figures 3A,B). We also observed no difference in dendritic volume between neurons infected with the various vectors (Figures 3C,D). An ANOVA revealed no significant effects of *Vector* [*F*_(2, 15)_ = 1.3, *p* = 0.29], *Branch order* [*F*_(2, 30)_ = 1.0, *p* = 0.37] or *Vector* × *Branch order* interaction [*F*_(4, 30)_ = 1.9, *p* = 0.13]. Therefore, manipulations of CREB function changed dendritic spine density without changing dendritic morphology.

### Spine morphology is not altered by CREB or CREB^S133A^ expression

**Figure 4:**
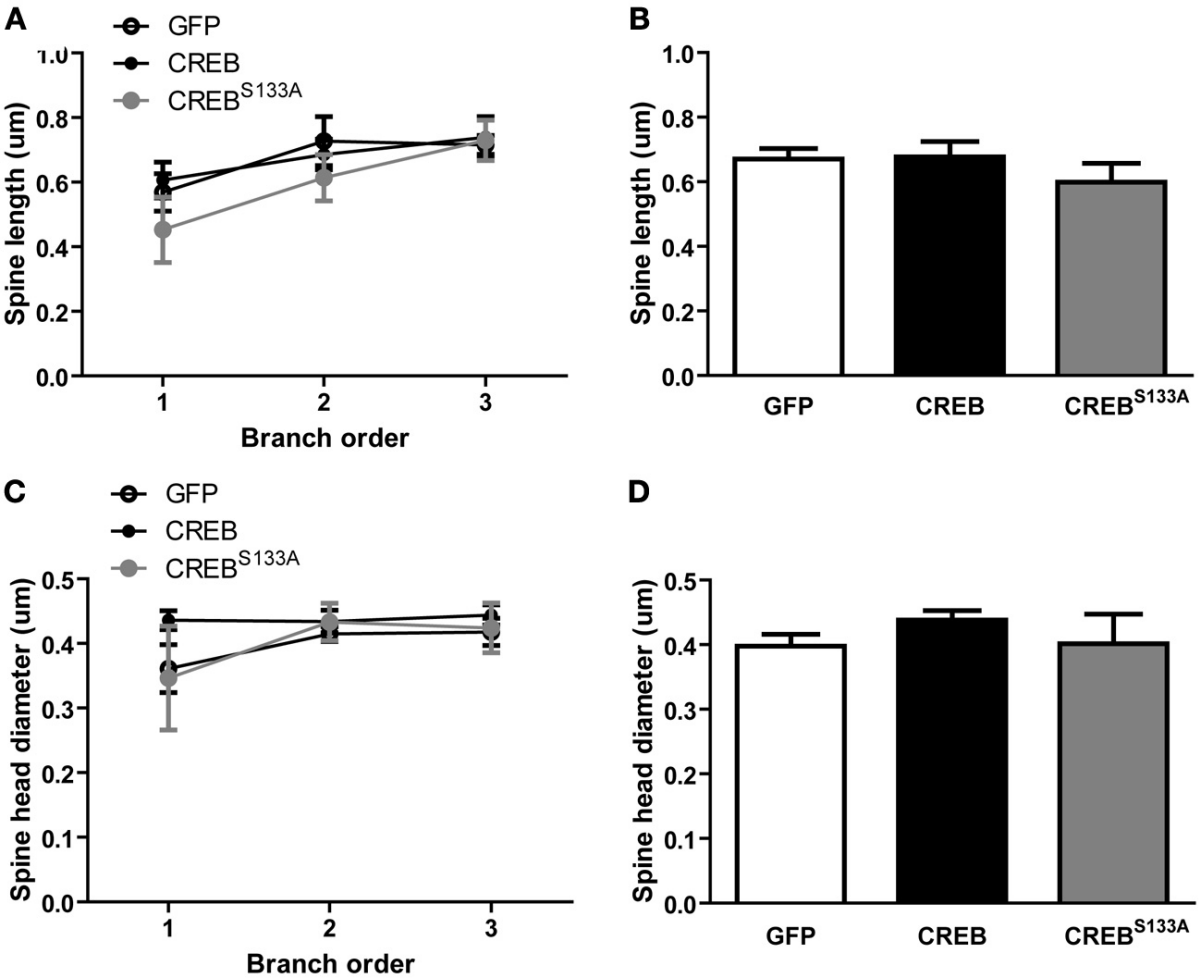
**CREB does not affect spine morphology. (A)** Spine length at each branch order and **(B)** across all branches was similar between LA neurons with GFP (*n* = 7), CREB (*n* = 6), or CREB^S133A^(*n* = 5) vectors. **(C)** Spine head diameter at each branch order and **(D)** across all branches did not differ between LA neurons with GFP (*n* = 7), CREB (*n* = 6), or CREB^S133A^ (*n* = 5) vectors.

Alterations in spine morphology have been correlated with changes in spine function (Matsuzaki et al., 2001, 2004) and increasing CREB may induce formation of silent synapses (Marie et al., 2005). Spines with large bulbous heads are thought to contain large post-synaptic densities (PSD) (Harris et al., 1992) whereas spines with small heads and long necks may contain silent synapses (Matsuzaki et al., 2001, 2004). Prompted by these observations, we analyzed whether manipulations of CREB function altered spine morphology in mice maintained in the homecage by measuring spine length and head diameter (see Figure 2B). Interestingly, spine length, regardless of vector, increased slightly with increasing branch order {Figures 4A,B; ANOVA showing no significant main effect of *Vector* [*F*_(2, 15)_ = 0.8, *p* = 0.45] or *Vector* × *Branch order* interaction [*F*_(4, 30)_ = 0.6, *p* = 0.63], but a significant main effect of *Branch order* [*F*_(2, 30)_ = 8.9, *p* = 0.0009]}. We next examined whether CREB manipulation influenced spine head diameter (widest distance of the spine head, see Figure 2B). We observed no difference between spine head diameter between vectors, but a small change per branch order across all vectors {Figures 4C,D no significant effect of *Vector* [*F*_(2, 15)_ = 0.7, *p* = 0.50] or *Vector* × *Branch order* interaction [*F*_(4, 30)_ = 1.0, *p* = 0.41] but a significant main effect of *Branch order* [*F*_(2, 30)_ = 4.2, *p* = 0.02]}. Therefore, although CREB manipulations produced changes in dendritic spine density, these were not accompanied by changes in dendrite or overall spine morphology.

## Discussion

Previously, we and others observed that neurons with relatively increased CREB function at the time of training seem to be competitively advantaged over neighboring neurons for allocation to a fear memory trace. Here we examined whether an increase in spine density at the time of training might mediate this competitive advantage. To this end, we examined the effects of manipulating CREB function on dendritic spine density at the time of training. We found that in mice taken directly from the homecage, neurons with CREB overexpression showed higher, while neurons with CREB^S133A^ showed lower spine density, than control infected neurons. These data are consistent with the notion that one factor that may determine neuronal allocation for memory formation is relative spine density.

The LA is a key brain region important in mediating fear and anxiety (Davis, 1992) and some studies implicate CREB in “emotional” behavior (Barrot et al., 2002; Pandey et al., 2003). It is possible, therefore, that CREB overexpression in a small population of LA neurons leads to a general increase in fear and/or anxiety. Disruption of CREB function either generally in the brain (Valverde et al., 2004) or specifically in the amygdala (Pandey et al., 2005) has been reported to increase anxiety-like behavior in mice. On the other hand, local CREB overexpression has been shown to enhance excitability of LA neurons without causing alterations in anxiety or locomotor activity (Viosca et al., 2009). In our experiments, before the presentation of the tone (pre-CS), freezing levels of mice overexpressing CREB or CREB^S133A^ did not differ from those overexpressing GFP. This ruled out the possibility that CREB or CREB^S133A^ might lead to alterations in general fear and anxiety.

Studies in the 1990s first implicated CREB in the formation of long-term memory (LTM) (Dash et al., 1990; Yin et al., 1994, 1995). Building on these important findings, we (Josselyn and Nguyen, 2005; Han et al., 2007, 2009; Cole et al., 2012), and others (Bourtchuladze et al., 1994; Kida et al., 2002) (Viosca et al., 2009; Zhou et al., 2009; Rexach et al., 2012), showed that decreasing CREB function disrupts, while increasing CREB function enhances the formation of many types of memory in mammals [but see Balschun et al. (2003)]. The role of CREB in fear memory has been extensively studied in rodents. Mice lacking α and δ isoforms of CREB (CREB^αδ^) showed impaired in LTM for both context and tone fear memories (Bourtchuladze et al., 1994). Similarly, CREB^comp^ mice, carrying one allele for the β isoform of CREB, showed deficits in LTM for context and tone fear memories (Gass et al., 1998). CREB^IR^ mice which express CREB^S133A^ in a temporally regulated manner have impaired context and tone fear memory following repression of CREB activity before training (Kida et al., 2002). Viral delivery of CREB into the amygdala using HSV enhanced LTM induced by massed training protocol in the fear potentiated startle paradigm in rats (Josselyn et al., 2001). CREB is thought to activate the transcription of target genes which ultimately serve as the building blocks for increasing the synaptic connections between neurons important for memory formation (Bartsch et al., 1998). It is interesting to note that CREB has also been implicated in human memory (Harum et al., 2001) and several human cognitive/memory disorders are linked to disruptions in the CREB signaling pathway (Josselyn and Nguyen, 2005). Together these data converge to indicate that CREB is critical for memory formation.

Previous studies have also established a possible role of CREB in maintaining spine number and morphology. Estradiol treatment in cultured hippocampal neurons led to increased phosphorylation of CREB which correlated with spinogenesis (Murphy and Segal, 1997). Enhancing CREB function upon expression of a constitutively active form of CREB (caCREB) in the CA1 region of hippocampus increased spine density in hippocampal neurons *in vivo* (Marie et al., 2005). CREB was also shown to regulate spine morphology in pyramidal neurons of the visual cortex (Suzuki et al., 2007). Expression of caCREB in organotypic hippocampal neurons increased spine density while decreasing CREB function by expression of a dominant negative CREB or a CREB-targeted shRNA inhibited spine formation (Impey et al., 2010). Consistent with this, increasing CREB function in hippocampal CA1 principal neurons restored the decrease in spine density and improved spatial memory in a mouse model of Alzheimer's disease (Yiu et al., 2011). Recent work has shown that CREB-induced excitability of LA neurons may be a potential mechanism for preferential recruitment of these neurons to the fear memory trace (Zhou et al., 2009).

Based on the previous work, we hypothesized that CREB's role in allocation of tone fear memory may be caused by its effect on regulation of spine density of LA neurons. The LA receives sensory (both tone and footshock) information directly from auditory cortex and thalamus (LeDoux et al., 1990; Campeau and Davis, 1995) and is thought to be the critical site for convergence of US and CS inputs in auditory fear conditioning experiments. Therefore, neurons with more dendritic spines may be preferentially activated by the CS and US convergence and become part of the memory trace.

Here, we observed that changes in CREB function alone were sufficient to change dendritic spine density, and that neurons with increased CREB function showed higher dendritic spine density. Furthermore we observed that neurons with higher CREB function were preferentially allocated to the memory trace. Because synapses and spines play a key role in neuronal information processing, changes in dendritic spine density or morphology of a neuron may affect synaptic function and local circuit organization. Along with other factors, such as changes in neuronal excitability (Zhou et al., 2009), changes in the synapse and spine number and morphology may influence neuronal spiking activity and play important roles in neuronal memory allocation.

### Conflict of interest statement

The authors declare that the research was conducted in the absence of any commercial or financial relationships that could be construed as a potential conflict of interest.
